# Leg length discrepancy in patients with slipped capital femoral epiphysis: 85 patients followed for mean 6 years

**DOI:** 10.3109/17453674.2013.795103

**Published:** 2013-05-31

**Authors:** Seung-Ju Kim, Tamir Bloom, Sanjeev Sabharwal

**Affiliations:** Department of Orthopaedics, New Jersey Medical School, Newark, NJ, USA.

## Abstract

**Background and purpose:**

Leg-length discrepancy (LLD) can be a sequela of slipped capital femoral epiphysis (SCFE). We tried to identify factors that affect the development of LLD following SCFE.

**Patients and method:**

We evaluated 85 patients who had been treated using percutaneous screw fixation. The average age of the patients at the time of surgery was 12 (8–16) years. The relationship of LLD and various clinical and radiographic parameters was evaluated: the degree of slip, articulotrochanteric distance (ATD), and articulotrochanteric distance difference (ATDD) (healthy side minus the side with SCFE). We assessed the relationship between ATDD and LLD based on scanogram.

**Results:**

The average LLD was 1.4 (0.1–3.8) cm at 6 (2–15) years postoperatively. 48 of 85 patients had an LLD of greater than 1 cm and 10 patients had an LLD of greater than 2 cm. There was a correlation between the magnitude of LLD and the severity of the slip. There was no statistically significant correlation between LLD and the stability of the slip, age, BMI, sex, or race. There was a significant correlation between LLD and ATDD.

**Interpretation:**

Patients with a high degree of slip are prone to develop clinically significant LLD. Although ATDD does not give the exact LLD, it can be used as a primary measurement, which should be supplemented with scanogram in cases of clinically significant differences in length.

Children with a slipped capital femoral epiphysis (SCFE) often have residual leg-length discrepancy (LLD). This is exacerbated further by premature physeal closure related to in situ fixation of the slip. As SCFE occurs before skeletal maturity, the LLD can increase over time. Inequality in leg length is commonly associated with compensatory gait abnormalities, and it may lead to degenerative arthritis of the lower extremity and lumbar spine in the middle to late adult years (Gurney 2002, Riad et al. 2010, Sabharwal and Kumar 2008). However, to our knowledge, the factors associated with development of postoperative LLD in patients with SCFE have not been thoroughly examined.

The purpose of the present study was to assess the magnitude of LLD associated with SCFE following in situ fixation, using plain radiography. We also wanted to determine the preoperative clinical factors that associated with the development of postoperative LLD. In addition, we examined the relationship between LLD as measured on a scanogram and the difference in articulotrochanteric distance (ATD) based on an anteroposterior (AP) pelvis radiograph in such patients.

## Patients and methods

### Study population

Approval from our institutional review board (registration number 0120110207) was obtained before the start of the study. All patients with a diagnosis of SCFE who had undergone in situ fixation from 1997 to 2010 at our department were identified. The following inclusion criteria had to be met before the patient was included in this retrospective study: in situ pinning had been performed and a postoperative scanogram or AP pelvis radiograph was available. Of the 146 patients who were identified, 10 patients were excluded because they did not have adequate follow-up radiographs. 51 patients who underwent bilateral operations and/or reoperations were excluded. 6 patients with postoperative complications (1 chondrolysis and 5 avascular necrosis) were also excluded because 4 of these patients underwent reoperations due to significant LLD and 2 had bilateral operations. The final study group consisted of 85 patients (85 hips). All 85 patients had postoperative AP pelvis radiographs and ATD measurements. 64 of the 85 patients had scanograms and both LLD and ATD measurements. 21 of the 85 only had ATD measurements. Prophylactic pinning was not routinely performed at our center, unless the patient had an underlying endocrinopathy or metabolic disorder that could predispose to SCFE.

Data regarding the patient’s age, sex, race, weight, height, side of involvement, duration of symptoms, stability of the slip, method and timing of treatment, duration of follow-up, and complications were collected from the medical records and review of previously taken radiographs. Using cutoff values of less than 30°, 30–60°, and greater than 60° for lateral head-shaft angulation (Siegel et al. 1991) as measured on the frog lateral view of the involved hip, 51 hips were classified as mild SCFE, 22 hips were classified as moderate SCFE, and 12 hips were classified as severe SCFE at initial presentation. The average age of the patients at the time of surgery was 12 (8–16) years. 46 of 50 male patients were more than 18 years of age and all the female patients were more than 16 years old. The minimum follow-up period was 2 years and the mean follow-up time was 6 (2–15) years.

### Surgical technique

Most procedures were performed by the senior author (SS). A 7.3-mm cannulated screw was inserted through a stab incision. For the patients with stable SCFE (Loder et al. 1993), in situ fixation was performed using a single screw, while in those with an unstable SCFE, 2 screws were used to obtain rotational stability. The patients were mobilized with partial weight bearing on the affected side for 4 weeks postoperatively. They were followed with serial radiographs every few months until skeletal maturity. None of the patients had postoperative infection or hardware failure.

### Radiographic assessment

**Figure 1.**
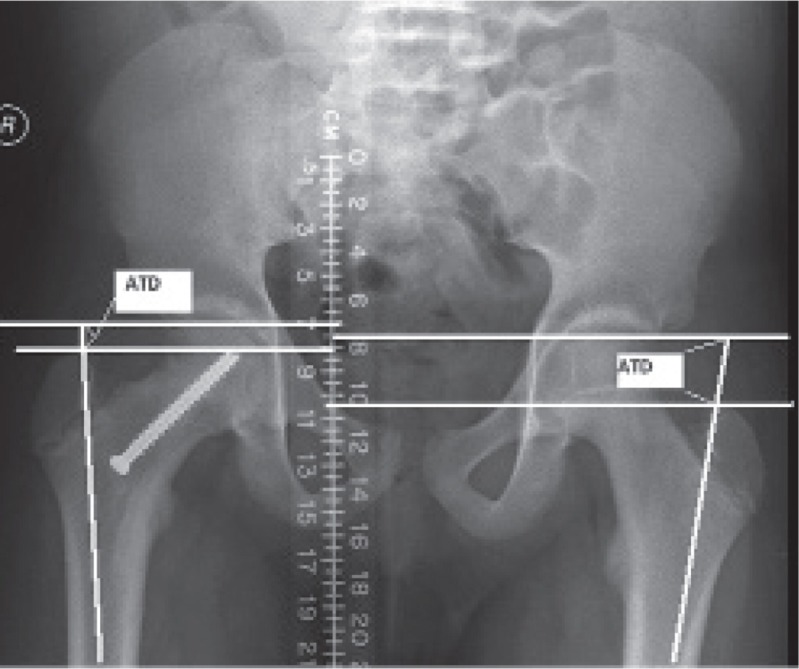
Measurement of the difference in articulotrochanteric distance (ATD).

The scanogram for measurement of LLD was done using computed radiography with the patient supine. The patient-to-tube distance was 101 cm. 3 separate AP images centered over the hip, the knee, and the ankle joints were made using three 35 × 43-cm computed radiography storage cassettes and were then distributed to the PACS workstation. The articulotrochanteric distance (ATD) was also measured from supine AP projections (Ordeberg et al. 1990). Perpendiculars to the longitudinal axis of the femoral diaphysis were drawn at the level of the proximal limit of the femoral head and at the tip of the greater trochanter (Figure 1). The ATD was given a positive sign if the proximal limit of the femoral head was situated proximally to the tip of the greater trochanter; otherwise it was negative. Articulotrochanteric distance difference (ATDD) was calculated as healthy side minus the side with SCFE. Correlation between LLD and ATDD was evaluated in the 64 patients who had both LLD and ATD measurements. A pediatric orthopedic surgeon (SJK) who was not involved in the clinical care of these patients made all of the measurements. Radiographs of 20 patients were randomly selected to assess intraobserver and interobserver reliability. Another pediatric orthopedic surgeon whose cases were included in this study (TB) independently measured the LLD and ATDD for the same 20 patients noted above. The radiographic parameters were tested for reproducibility using Pearson correlation coefficients. The correlation coefficients ranged from 0.927 to 0.981 for intraobserver reliability and from 0.913 to 0.963 for interobserver reliability. Mean absolute difference (MAD) in mm for measurements of LLD was 1.7 mm (95% CI: 1.3–2.2).

### Statistics

The difference between the initial LLD and LLD at the latest follow-up was analyzed using paired t-test. Effects of patient sex, race (negroid or others) and stability on LLD and ATDD was assessed with the Mann-Whitney U-test. Comparison of LLD and ATDD among the groups with different slip severity (mild, moderate, severe) was analyzed using the Kruskal-Wallis test for nonparametric data. Multiple linear regression analysis was used to determine the relationship between LLD and independent variables such as slip angle, age, and BMI. The relationship between LLD and ATDD was determined using linear regression analysis. A p-value of < 0.05 was considered to be significant. We used SPSS software version 12.0.

## Results

The mean values of LLD at the time of surgery (in situ pinning) and latest follow-up were 1.0 cm (SD 0.6; 95% CI: 0.2–1.8) and 1.4 cm (SD 0.8; CI: 0.3–2.5), respectively. The mean values of ATDD at the time of surgery and latest follow-up were 0.8 cm (SD 0.5; CI: 0.2–1.4) and 1.0 cm (SD 0.7; CI: 0.2–1.8), respectively. 48 patients had LLD greater than 1 cm and 10 patients had LLD greater than 2 cm. There was a significant difference between the initial LLD and the LLD at latest follow-up (p = 0.02).

As the slip severity increased, the LLD and ATDD increased (p < 0.001 and 0.02, respectively), based on multivariate analysis. We did not find a statistically significant relationship between LLD or ATDD and age at surgery. Furthermore, there was no significant relationship between LLD or ATDD and BMI (Table).

**Table:** Leg-length discrepancy (LLD), articulotrochanteric distance difference (ATDD), and p-values according to different factors based on bivariate analysis

Factor	No. of patients for LLD	Final LLD (cm) and 95% CI	p-value	No. of patients for ATDD	Final ATDD (cm) and 95% CI	p-value
Sex						0.4
Male	34	1.4 (0.3–2.5)		50	1.0 (0.2–1.8)	
Female	30	1.3 (0.2–2.4)		35	0.9 (0.2–1.6)	
Race						0.6
Negroid	46	1.4 (0.2–2.6)		61	1.1 (0.2–2.0)	
Others	18	1.2 (0.4–2.0)		24	1.0 (0.2–1.8)	
Severity of slip			< 0.001			0.02
Mild	41	1.0 (0.2–1.8)		51	0.7 (0.2–1.2)	
Moderate	13	1.4 (0.2–2.6)		22	1.0 (0.2–1.8)	
Severe	10	1.9 (0.3–3.5)		12	1.4 (0.2–2.6)	
Stability of slip						0.2
Stable	53	1.3 (0.2–2.4)		72	1.0 (0.2–1.8)	
Unstable	11	1.5 (0.2–2.8)		13	1.1 (0.1–2.1)	

**Figure 2.**
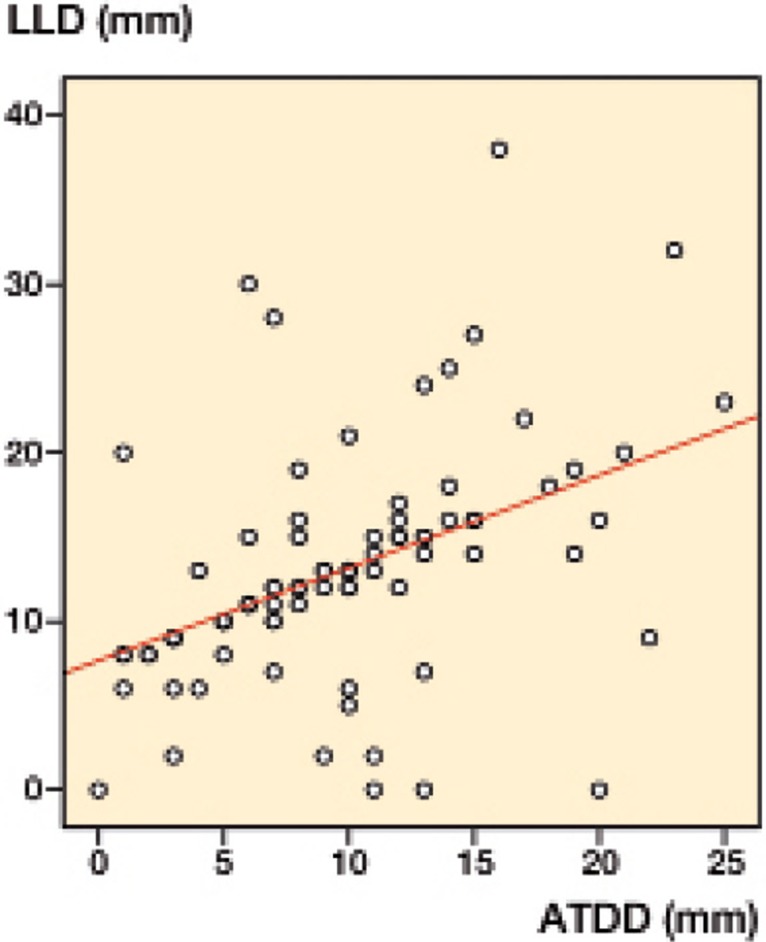
Regression analysis of 64 patients who had both limb-length discrepancy (LLD) measurements and articulotrochanteric distance (ATD) measurements, showing the relationship between LLD determined with the use of a scanogram and articulotrochanteric distance difference (ATDD) based on pelvic radiographs.

Linear regression analysis showed that there was a correlation between the measured LLD and ATDD (p < 0.001) (Figure 2).

## Discussion

Overall, we found that in our cohort of patients with SCFE who underwent surgical stabilization, the LLD averaged 1.4 cm. Furthermore, 10 of the 85 patients had more than 2 cm of shortening of the affected extremity.

As anticipated, our data support the notion that the magnitude of LLD is closely related to the severity of SCFE. The demographic features of our patient population such as age, sex, BMI, race, stability, and slip severity were similar to those reported by others (Hagglund et al. 1984, Loder et al.1993, Loder 1996, Song et al. 2004, DeLullo et al. 2007). Despite the increasing evidence that obesity is associated with the occurrence and severity of SCFE (Kelsey et al. 1972, Poussa et al. 2003, Bhatia et al. 2006), we did not find any influence of the patient’s BMI on LLD or ATDD. Loder et al. (1993) emphasized the importance of physeal stability at presentation by correlating it with clinical outcome. We were unable to find any significant relationship between the stability of the slip and LLD. This may have been caused by the limited number of patients with unstable SCFE in our series, and with the inadvertent reduction that may have occurred during surgical stabilization of such slips, the severity of the slip could have been reduced.

LLD at skeletal maturity may often be more pronounced when SCFE occurs at an earlier age, since in situ pinning can induce premature fusion of the affected physis. In the present study, LLD increased from 1.0 cm to 1.4 cm with longer follow-up. Nevertheless, we observed that the patients who underwent in situ pinning at an older age often had an increase in LLD compared to the patients who underwent operation at a younger age. A possible reason for this finding may be that the angle of the slip increased (p = 0.08) according to age, as a result of the increased mechanical load across the proximal femoral physis.

The ATD has also been shown to be a useful marker for studying growth disturbances of the proximal femur in patients with SCFE. The ATD is normally positive, with a mean value of about 20 mm (Ordeberg et al. 1990). Reduction of ATD is often seen with premature closure of the subcapital growth plate or conditions associated with altered morphology of the proximal femur such as growth disturbances, coxa vara, and SCFE. We found a significant relationship between the LLD and ATDD. These findings support the use of AP pelvic radiography as the initial imaging study to assess LLD by measuring the difference in ATD between the two sides in patients with SCFE. This approach can possibly reduce the expense and radiation exposure while still allowing reasonably accurate estimation of the LLD, compared to the use of additional imaging studies for the assessment of limb-length discrepancy in patients with SCFE.

This study had some limitations. Firstly, to represent the final LLD, all patients should have attended a radiographic evaluation after physeal closure. In our series, the postoperative mean age of 6 (2–15) years meant that skeletal maturity was not assured in some of these patients. However, the mean age at the time of the final scanogram was 18.6 years, which is generally beyond the timing of physeal closure. 46 of 50 male patients were over 18 and all the female patients were over 16. Furthermore, most children with SCFE are obese (Poussa et al. 2003), which is associated with premature physeal closure. Secondly, there were a limited number of patients with unstable SCFE.

In summary, we found the largest amount of LLD in patients with a high degree SCFE, which would seem intuitive. However, the factors associated with development of postoperative LLD in patients with SCFE have not been well studied. We also found that the difference in the ATD as measured on an AP radiograph of the pelvis can be a surrogate for assessing LLD. These findings can influence the clinical decision making when managing children with SCFE.
